# Emergence of category-level sensitivities in non-native speech sound learning

**DOI:** 10.3389/fnins.2014.00238

**Published:** 2014-08-08

**Authors:** Emily B. Myers

**Affiliations:** ^1^Department of Speech, Language, and Hearing Sciences, University of ConnecticutStorrs, CT, USA; ^2^Department of Psychology, University of ConnecticutStorrs, CT, USA; ^3^Haskins LaboratoriesNew Haven, CT, USA

**Keywords:** speech perception, phonetic category, second language acquisition, inferior frontal gyrus, superior temporal gyrus

## Abstract

Over the course of development, speech sounds that are contrastive in one's native language tend to become perceived categorically: that is, listeners are unaware of variation within phonetic categories while showing excellent sensitivity to speech sounds that span linguistically meaningful phonetic category boundaries. The end stage of this developmental process is that the perceptual systems that handle acoustic-phonetic information show special tuning to native language contrasts, and as such, category-level information appears to be present at even fairly low levels of the neural processing stream. Research on adults acquiring non-native speech categories offers an avenue for investigating the interplay of category-level information and perceptual sensitivities to these sounds as speech categories emerge. In particular, one can observe the neural changes that unfold as listeners learn not only to perceive acoustic distinctions that mark non-native speech sound contrasts, but also to map these distinctions onto category-level representations. An emergent literature on the neural basis of novel and non-native speech sound learning offers new insight into this question. In this review, I will examine this literature in order to answer two key questions. First, where in the neural pathway does sensitivity to category-level phonetic information first emerge over the trajectory of speech sound learning? Second, how do frontal and temporal brain areas work in concert over the course of non-native speech sound learning? Finally, in the context of this literature I will describe a model of speech sound learning in which rapidly-adapting access to categorical information in the frontal lobes modulates the sensitivity of stable, slowly-adapting responses in the temporal lobes.

## Introduction

Phonetic categories, the basic perceptual units of language, are defined over distributions in acoustic space. For any phonetic category (e.g., /d/) there will be a range of acoustic tokens that will all be computed as acceptable members of a given phonetic category. To take a classic example, voiced and voiceless stops (e.g., /d/ vs. /t/) are primarily distinguished in initial position by the acoustic/articulatory parameter known as voice onset time, or VOT. For a native English speaker, VOTs less than about 30 ms are heard as /d/ sounds and those greater than 30 ms are perceived as /t/ sounds. The process of learning phonetic categories requires that the listener learn the boundaries of this acoustic space in order to understand how any given acoustic token maps to the phonology of his/her native language. To take the example given above, the English-learning child will learn that the voicing boundary falls at about 30 ms VOT in her language, but the Spanish-learning child will learn a boundary at about 0 ms VOT (Lisker and Abramson, 1964). This learning process is complicated by the fact that phonetic categories are typically defined by multiple acoustic parameters (e.g., VOT, vowel length, closure duration, burst amplitude). In this sense, we may think of the process of learning phonetic category boundaries as one of defining a hyperplane through multi-dimensional acoustic space.

In theory, all that is necessary for successful phonetic processing is the discovery of the location of phonetic boundaries in acoustic space. However, human speech perception is more complex than this. Over the course of development, acoustic differences that are contrastive in the child's native language become perceived as more distinctive, while those that are non-contrastive (i.e., they fall within the same phonetic category) become perceived as less distinctive (Eimas et al., 1971; Werker and Tees, 1999; Polka et al., 2001; Best and McRoberts, 2003; Kuhl et al., 2008). This perceptual pattern, namely excellent discrimination of items that fall between categories in the face of poor discrimination of items within phonetic categories, is referred to as categorical perception (Liberman et al., 1957). Through early childhood, this trajectory continues, with native-language contrasts becoming perceived more categorically and non-native contrasts becoming less categorical between ages 2 and 6 (e.g., Burnham et al., 1991). By the time listeners reach adulthood, many phonetic categories are perceived categorically, and as such the mature phonetic processing system is not only sensitive to the boundaries of phonetic space, but exhibits perceptual warping such that certain portions of that space are easier to discriminate than others.

**Figure 1:**
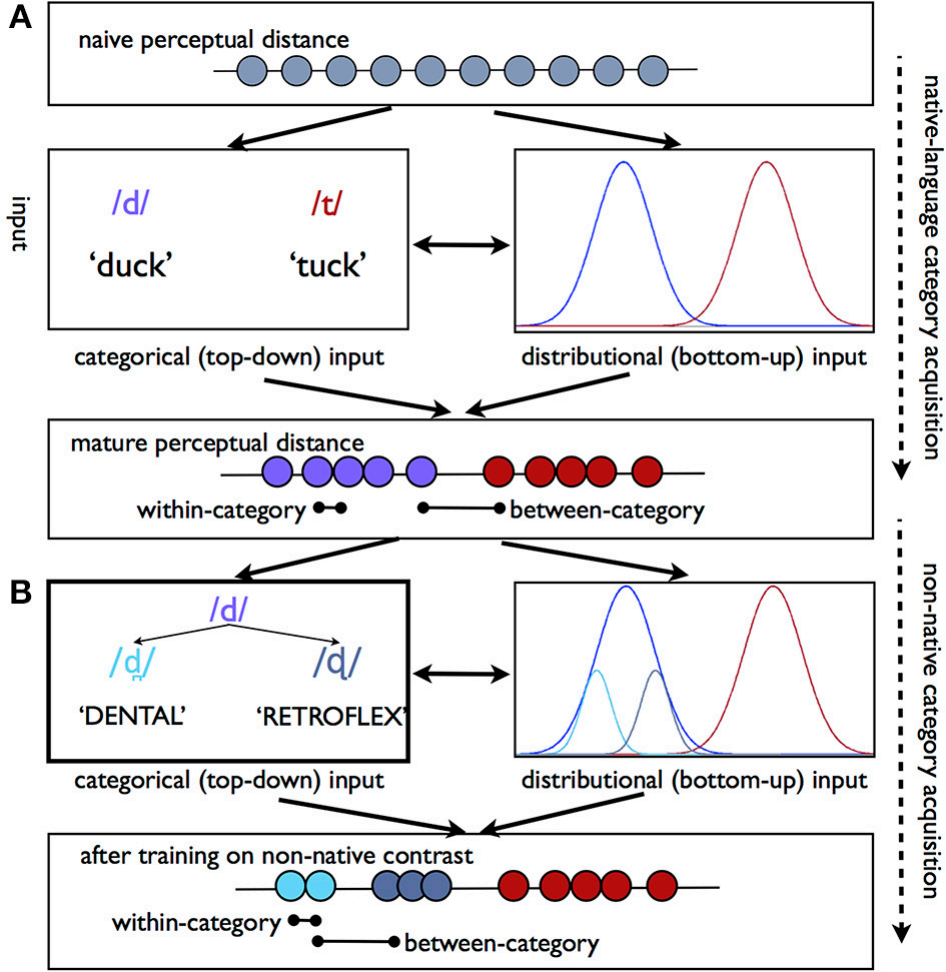
**Perceptual warping as a consequence of phonetic category learning**. **(A)** Schematic of the process by which categorical perception emerges through development. Top line reflects the naïve perceptual distance between tokens along an arbitrary acoustic-phonetic continuum. Over the course of development categorical information (e.g., the use of tokens to refer to minimal pairs) and the statistical distribution of tokens in acoustic-phonetic space (e.g., more tokens are heard that fall near the center of the phonetic category) converge to warp perceptual sensitivities such that between-category contrasts are more perceptually distinct than within-category contrasts. **(B)** Non-native speech sound training paradigms primarily rely on categorical-level cues (e.g., explicit feedback), to reshape existing sensitivities. In this particular example, a listener must learn that two non-native sounds which are typically perceived as variants of /d/ correspond to different categories. This type of learning situation presents a particular challenge to the adult learner, given that the perceptual distance between these tokens in the mature listener is collapsed. Learning may proceed either via the top-down route, (left), or via passive exposure to statistical regularities in the input (right), or both. Over time, this information likewise results in differences for within- vs. between-category perceptibility.

It is a matter of significant debate as to how categorical perception emerges. One proposal is that the statistical distribution of phonetic tokens in acoustic-phonetic space may provide sufficient information to reshape perceptual sensitivities even before functional phonetic categories have developed in the learner (Kuhl et al., 1992; Guenther and Gjaja, 1996; Maye et al., 2002, 2008). This view stems from the observation that the speech tokens that listeners are exposed to are not evenly distributed in acoustic space. For instance, the listener will hear many more examples of /t/ with a VOT near 60 ms will than with a VOT of 120 ms, although both are considered to be members of /t/ category (Figure 1A). Some evidence suggests that infant and adult listeners alike may be able to take advantage of distributional/statistical information in order to amplify acoustic distinctions that fall between different distributions and minimize those within the distribution (Maye et al., 2002, 2008; Hayes-Harb, 2007; Emberson et al., 2013). Crucially, this perceptual reshaping can happen even when listeners know nothing about the functional use of phonetic categories—that is, when listeners are only passively exposed to the input, and never hear speech sounds used referentially.

Nonetheless, young and old learners alike are exposed to additional sources of information regarding the sounds that are contrastive in their language. The use of phonetic categories to refer to different visual objects has been shown to result in better discrimination of those sounds (Yeung and Werker, 2009), and the appearance of different sounds in different lexical contexts may have a similar effect (Feldman et al., 2013). Ultimately, it is clear that the language learner must eventually learn the phonology of his or her own language. This sort of top-down information may continue to reshape perceptual sensitivities to these same sounds as the language user matures (Figure 1A). Given that the warping of perceptual space seen in adults may have arisen both as a consequence of passive, bottom-up data derived from the statistical distribution of tokens in the input, as well as the acquisition of functional, category-level information about the phonology of one's language, it is challenging to attribute behavioral and neural patterns we observe in adult phonemic perception to either bottom-up sensitivities to the acoustic input or top-down knowledge of phonetic category status.

This obstacle is particularly evident when discussing the neural systems that are responsive to phonetic category identity. For instance, if it is the case that statistical/distributional information in the signal is sufficient to guide the emergence of phonetic category identity, neural structures that are responsive to phonetic category structure may be those that have formed as a function of these bottom-up properties of the signal rather than as a response to the functional, linguistic use of phonetic categories. To the extent to which we believe passive mechanisms may also be sufficient to reshape sensitivities to complex acoustic information in auditory and auditory association cortex (Pallier et al., 1997; Zhou and Merzenich, 2007), mature, native language neural sensitivity may in large part reflect these bottom-up mechanisms.

In order to develop plausible hypotheses about the nature of phonetic category formation in adult, non-native acquisition, it is first important to discuss current evidence regarding the neural processing of native-language phonetic category structure.

## Native language phonetic category structure in the brain

It has been well established that the bilateral superior temporal lobes are preferentially responsive to intelligible speech sounds compared to identifiable non-speech sounds (e.g., Belin et al., 2000, 2002), and compared to acoustically-matched sounds which are unintelligible as speech. (Okada et al., 2010; Evans et al., 2013). Recent evidence from direct cortical recording has revealed populations of neurons that code for dimensions of the phonetic inventory, including place of articulation and manner of articulation, showing that the human temporal lobes are well-equipped to distinguish between the sounds of speech (Chang et al., 2010; Mesgarani et al., 2014). What is less clear is the extent to which these systems are specifically tuned to native-language contrasts or whether they show a more general sensitivity to, or preference for, many classes of speech sounds (for a more complete review, see Turkeltaub and Branch Coslett, 2010).

In order to answer this question, the review below is restricted to evidence in which the neural response reflects specific sensitivity to the internal structure of native-language speech categories. In particular, studies which show different responses to variability within and between categories can be said to show this kind of sensitivity.

### Mid-to-posterior superior temporal gyrus tuning to native-language category structure

As a seat of complex acoustic processing, the bilateral temporal lobes play a primary role in processing the auditory details of the speech signal. Evidence suggests that there is a gradient of sensitivity along the temporal lobe from finer-grained acoustic processing near Heschl's gyrus (HG) to increasing specificity in tuning to one's native language as the processing stream flows in both the anterior and posterior directions along the STG/STS. In particular, middle portions lateral to HG have been shown to respond to native speech sounds compared to well-controlled non-speech sounds (Liebenthal et al., 2005; see Turkeltaub and Branch Coslett, 2010; DeWitt and Rauschecker, 2012 for meta-analyses). In contrast, regions including middle-STG territory lateral to HG and extending posterior along the STG/STS have been more tightly linked to phonological processing, and in particular have been shown to be sensitive to phonetic category structure. For instance, the bilateral superior temporal gyrus and superior temporal sulcus (STG and STS) are sensitive to how typical a speech sound is a member of its phonetic category (Guenther et al., 2004; Myers, 2007). This gradient response reflects the non-uniform structure of phonetic categories, suggesting that the temporal lobes are tuned to the internal perceptual structure of native-language categories, and are not merely sensitive to all speech sound dimensions.

The sensitivity of left posterior temporal areas in the perception of contrasts between- and within-category is supported by a series of studies using repetition suppression or habituation designs. While these studies differ in their details, all share a design in which a repeated presentation of a phonetic stimulus is followed by either an identical stimulus or a change in stimulus. Neural sensitivity to changes between and within the category are assessed by comparing activation for “change” trials to “repeat” trials. More categorical responses, as reflected by selective sensitivity to between-category compared to either repeated or within-category contrasts, were found in the left supramarginal gyrus, and in left posterior superior temporal sulcus (Joanisse et al., 2007; Myers et al., 2009).

Evidence that the temporal lobes respond to native-language contrasts also comes from the mismatch negativity paradigm. Larger MMN responses are seen to deviant tokens which cross a phonetic category boundary than those that change within the category (Phillips, 2001). Of interest, the MMN source is thought to arise from bilateral temporal cortex, shows greater left-lateralization for native language contrasts (see Naatanen et al., 2007 for review; Zevin et al., 2010), and MMN responses over the left temporal lobe are larger to phonetic than non-phonetic contrasts when employing direct cortical recording (Molholm et al., 2014), particularly in or near the STS. This MMN response is not restricted to temporal lobes however; the MMN response is thought to have a secondary source in left prefrontal cortex (Paavilainen et al., 2003, see further discussion of frontal contributions in section “Left inferior frontal involvement in categorical responses to native-language contrasts”).

Discussion above has been limited to studies which specifically show differences in responsiveness to within vs. between-category contrasts. Nonetheless, converging evidence from other types of designs suggests that posterior portions of the left STG/STS are responsive to the category identity of native-language speech sounds (e.g., Desai et al., 2008; Chang et al., 2010; Liebenthal et al., 2010; Mesgarani et al., 2014). Of interest, speech category sensitivity in temporal regions is not limited to purely perceptual paradigms but it is also evident in auditory feedback for speech motor control. In particular, when speakers receive perturbations to auditory feedback that fall near the phonetic category boundary, greater compensation is seen in the speech production response, with concomitant greater activation for near-boundary compared to far-boundary shifts in the bilateral posterior STG (Niziolek and Guenther, 2013). Taken together, these results suggest that the posterior superior temporal lobes, particularly on the left, show fine-grained tuning to the acoustic properties of one's native language, with greater (or perhaps selective) neural sensitivity to acoustic distinctions that result in a change in phonetic category. It is of note that responses in the posterior STG/STS are not driven solely by bottom-up characteristics of the acoustic signal, but are also modulated by shifts in phonetic category boundary, and by changes in the perceptual status of the stimulus (e.g., non-speech to speech) (e.g., Desai et al., 2008; Gow et al., 2008; Myers and Blumstein, 2008).

### Left inferior frontal involvement in categorical responses to native-language contrasts

While the temporal lobes no doubt shoulder much of the burden in processing the sounds of speech, evidence suggests that left prefrontal cortex also plays a role in the computation of phonetic identity. In two passive repetition suppression studies, responses to category-level information (e.g., greater responses to between-category than within-category shifts, yet no difference between within-category and repeated trials) were seen in premotor areas (Chevillet et al., 2013), and in an “invariant” response in the precentral gyrus and pars opercularis (Myers et al., 2009). Pre-motor areas which had been identified as sensitive to between-category changes showed significant task-related functional connectivity during passive listening to sites in the posterior temporal lobes (Chevillet et al., 2013), which led to the interpretation that phonetic category computations rely on forward projections between the temporal and frontal lobes along the dorsal route (Hickok and Poeppel, 2004). A recent analysis by Lee et al. (2012) examined category-level sensitivity of several brain regions using new data in which participants passively listened to syllables along a ba—da continuum as well as using existing data from a repetition suppression paradigm (Raizada and Poldrack, 2007). In this study, the authors employed a moving searchlight technique with whole-brain multi-voxel pattern analysis (MVPA, Kriegeskorte et al., 2006) to search for clusters of voxels in which the patterns of activation could discriminate between two different phoneme categories (da vs. ba). Sensitivity to category-level information was seen in the left pars opercularis and pre-supplementary motor region as well as in the left superior temporal lobe. Converging evidence from studies in which cortical processing is disrupted using TMS also points to a role for frontal structures in computing category membership: stimulation of motor cortex sites slightly alters categorical perception in phoneme categorization and discrimination tasks (Mottonen and Watkins, 2009; D'Ausilio et al., 2012).

What is less clear is the precise role or roles of these frontal structures, which may indeed constitute functionally distinct sub-regions within the frontal lobes. The implication of premotor areas has led to the hypothesis that articulatory codes for speech may be activated to either guide perceptual hypotheses generated in the temporal lobes, or, more radically, to act as the contents of the abstract speech sound category (Liberman and Mattingly, 1985). At the same time, the influence of frontal areas may not be limited to access to articulatory information, nor, indeed, is category-sensitive activation limited to premotor cortex. Anterior to premotor cortex, regions in Broca's homolog have been found to be sensitive to category-level information in a domain-general sense, and evidence from single-cell recordings in non-human primates suggests that invariant responses to category membership may arise in frontal areas (e.g., Freedman et al., 2001). As such, the involvement of frontal areas may not reflect motor-related activity, but may reflect access to a more abstract category representation. In general, these results suggest that a complex of information arising from prefrontal regions generally may guide perception (Davis and Johnsrude, 2007; Liebenthal et al., 2013).

At the same time, the role of frontal structures in speech intelligibility “in the wild” has been questioned (Hickok and Poeppel, 2007; Hickok et al., 2011). It has been observed that lesions to left inferior frontal areas need not impair explicit decisions of phonetic category identity, and rarely create errors in phonemic perception (Basso et al., 1977; Rogalsky et al., 2011), and that while stimulation of premotor sites may impair categorization decisions, there is no evidence of deficits in comprehension as a result of such stimulation (Krieger-Redwood et al., 2013). Engagement of frontal structures for speech perception has been especially observed in the presence of ambiguity or noise in the signal (Binder et al., 2004; D'Ausilio et al., 2012), and as such frontal areas are argued to be peripheral to processing the sounds of speech. Some (D'Ausilio et al., 2012) while agreeing that frontal involvement for perception seems especially important in the context of noise in the signal, point out that noisy signals and imperfect productions are actually the norm rather than the exception in the typical language environment, and that we should resist the temptation to view frontal influences in speech perception as epiphenomenal. As such the types of activation patterns observed in studies of categorical perception can be accommodated by assuming that frontal structures are consulted in less optimal listening conditions.

**Figure 2:**
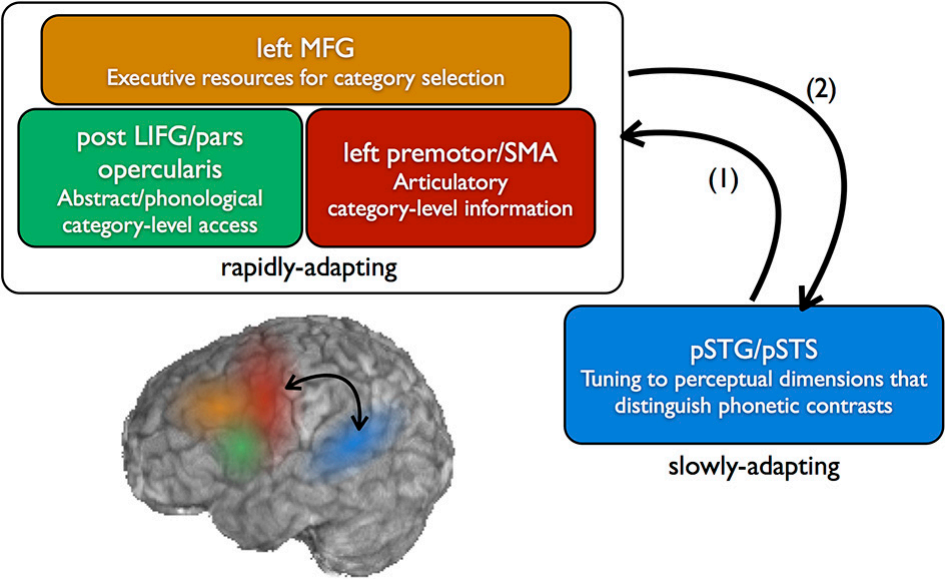
**Neural systems for the perception and learning of speech sound categories**. Fine-grained sensitivity to acoustic dimensions that distinguish native speech sounds (e.g., VOT) is found in the posterior superior temporal gyrus (pSTG) and superior temporal sulcus (STS), which includes preferential sensitivity to speech categories, but, to a lesser degree, also sensitivity to within-category variation. In perception, sounds which are not well-categorized by this tuning (e.g., ambiguous sounds) feed forward to categorical-level coding in the frontal lobe (1). For non-native category learning which relies on top-down feedback, category sensitivities may emerge first in the frontal lobe, then feed back to posterior temporal areas to guide long-term changes in perceptual sensitivity (2). This frontal-to-temporal feedback corresponds to the top-down learning route shown in the bottom left portion of Figure 1.

Whether the codes accessed in the inferior frontal lobes are articulatory or abstract in nature, evidence suggests that coding in the left prefrontal areas is more categorical than that represented in the temporal lobe. This suggests an architecture whereby fine-grained acoustic-phonetic details of the speech stream are processed in the left STG/STS, and this information is then projected forward to prefrontal regions to consult with categorical-level codes in a complex of frontal areas (Figure 2).

## Non-native phonetic category acquisition: a case of functional plasticity

As discussed above, the mature language learner comes to the second-language learning process with a set of pre-established perceptual sensitivities which may either facilitate or hinder the acquisition of a new category. In particular, to learn a new phonetic contrast which falls within the acoustic territory occupied by native language sounds, the listener must learn to either (a) shift an existing phonetic category boundary, as in the case of an English speaker learning the a VOT boundary which corresponds to the Spanish d/t contrast, or (b) divide an existing phonetic category into two, as in the case of the English listener learning to perceive the Hindi dental vs. retroflex stop contrast (Figure 1B). This latter scenario seems particularly challenging, as an entire native-language architecture has developed which prevents the listener from perceiving distinctions within the phonetic category.

By the time adulthood is reached, one's sensitivities to native-language phonetic categories have reached a stability point. In fact, non-native categories, particularly those that fall within an existing native-language category, are notoriously difficult to acquire in adulthood (Best et al., 1988). The fact that even motivated adults can struggle to distinguish certain non-native contrasts has led to conclusion that there is a critical period for phonetic category learning. This critical period may result from losses in neuroplasticity which prevent the adult listener from altering perceptual sensitivities in order to accommodate these into native language category structure (Pallier et al., 1997).

Nonetheless, with sufficient training, many individuals are able to learn to perceive non-native contrasts (Logan et al., 1991; Lively et al., 1993; Bradlow et al., 1997; Golestani and Zatorre, 2009), with some learners achieving native-like proficiency. Individuals who learn to speak a second language “in real life” (as opposed to in laboratory training conditions) have multiple sources of information which can guide the formation of new phonetic categories. Much as infants may be able to use information regarding the statistical distribution of phonetic tokens in acoustic space to reshape sensitivities, adults who are exposed to a non-native language will likely hear the same kinds of distributional information, whether they are able to take advantage of it or not (Figure 1B). Crucially for the adult learner, top-down information about phonetic category identity, either in the form of referential information (e.g., using two sounds to refer to two different words) or even through explicit classroom instruction, is often very salient in the environment. Unfortunately, almost all studies regarding the emergence of non-native phonetic sensitivity in the brain have used training paradigms where top-down information about category identity is provided to participants (see left side of Figure 1B). As such, we can draw limited conclusions regarding the emergence of neural sensitivities to non-native contrasts via more passive, bottom-up mechanisms in which listeners capitalize on distributional properties of the input.

### Perceptual warping from non-native category training

Before considering the neural structures that are sensitive to phonetic category training, it is first important to assess whether top-down (e.g., categorization) training results in a perceptual pattern that resembles native language perception. As discussed above, acquisition of a native-language contrast appears to involve not only learning the boundary between categories, but also results in changes in perception of acoustic contrasts within and between these categories. Given that the types of training paradigms used in many studies bear a scant relationship to the authentic language acquisition environment, it would not be surprising to find that participants might successfully be able to complete a categorization task using non-native stimuli (that is, learn the location of the category boundary) while showing no difference in the relative perceptibility of between and within-category contrasts. Fortunately, converging evidence suggests that training participants on category-level information results in changes in discriminability of tokens across the trained continuum. Studies investigating training on the /l/ vs. /r/ contrast in native-Japanese listeners (McCandliss et al., 2002), and the Hindi dental vs. retroflex stop contrast (/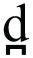/ vs./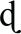/) in English listeners (Golestani and Zatorre, 2009) show that training on categorization tasks transfers to discrimination tasks, and specificity of the discrimination peak appears to be closely linked to both the location of the learned category boundary for each participant as well as to the relative success of each listener in acquiring the new contrast (Guenther et al., 1999; Wade and Holt, 2005; Golestani and Zatorre, 2009; Swan and Myers, 2013).

### Functional brain changes resulting from non-native category training

When adults learn a non-native contrast, either via explicit category training, or from more naturalistic experience, brain structures which show specific sensitivity to native language contrasts must somehow reshape responses in order to accommodate a new categorical division of acoustic space. In general, we may ask whether the same neural resources are recruited for non-native speech sound perception following training as are implicated for native-language perception. Non-native phonetic training often takes the form of categorization training on either syllables or minimal pairs with explicit feedback to participants, often using a perceptual fading design, in which participants initially categorize maximally distinct tokens, then proceed to finer distinctions in a stepwise fashion (Golestani and Zatorre, 2004; Liebenthal et al., 2010; Myers and Swan, 2012). In this situation the availability of category-level information can be said to be at its maximum, as participants receive feedback regarding the accuracy of the categorical decision. When examining task-related activation before and after training, a wide network of regions are recruited, including bilateral temporal and left inferior frontal structures (Callan et al., 2003; Golestani and Zatorre, 2004) which show greater task-related activation to non-native sounds after compared to before training. Concordant evidence using a similar training paradigm yielded greater activation for non-native categorization post-training in a series of frontal regions and left inferior parietal regions (Ventura-Campos et al., 2013). Given the explicit nature of the categorization task, these studies are vulnerable to the criticism that the activation in inferior frontal regions is related to the metalinguistic task, rather than to the perception of phonetic category differences *per se* (see Section, “Left inferior frontal involvement in categorical responses to native-language contrasts,” above).

Nonetheless, a study from our lab supported the involvement of a separate set of frontal structures, namely the left and right middle frontal gyri in categorical perception of learned speech sounds (Myers and Swan, 2012). In this study, participants were trained to categorize a three-way phonetic continuum (voiced stops ranging from dental to retroflex to velar place of articulation: /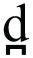/vs./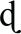/vs./g/) according to two different boundary locations, with one group trained to place the category boundary between the dental and retroflex tokens, and a separate group trained to place the category boundary between the retroflex and velar tokens. Participants were trained over two sessions, and neural sensitivity post-training was assessed using an short-interval habituation design which did not require participants to categorize speech sounds (see Joanisse et al., 2007; Myers et al., 2009). Despite the fact that the task required no judgments of phonetic category identity during scanning, activity in the bilateral middle frontal gyri reflected differential sensitivity to between vs. within-category contrasts according to the training of the participants. Of interest, no difference in activation for between vs. within-category contrasts was seen in the temporal lobes, suggesting that differential responsiveness to learned category structure need not rely on retuning of sensitivities in the temporal lobe.

Support for the involvement of inferior frontal regions for non-native category learning can be seen in other passive paradigms. An analysis of resting-state functional data before and after intensive (one day) and distributed (six sessions) of non-native category training suggested that a decrease in degree of functional connectivity between two regions of interest in the left frontal operculum and left superior parietal lobule was significantly correlated with participant accuracy (Ventura-Campos et al., 2013). To unpack this result further, this suggests that individuals who were more successful in learning the non-native contrast showed a decrease in the degree of coherence between frontal and parietal structures, perhaps reflecting a decreased reliance on the frontal-to-parietal connection over the course of learning.

Nonetheless, training-related activity is not exclusive to these frontal regions. A series of training studies have shown significant involvement of temporal structures in sensitivity to trained speech and complex non-speech sounds. Liebenthal et al. (2010) trained participants over four sessions to identify non-speech sounds which resembled speech sounds in their spectral and temporal properties. Activation in the left posterior STS increased for trained non-speech sounds following training, with additional small clusters in left inferior frontal areas. Similarly, Leech et al. (2009) used an implicit training method which paired complex non-speech sounds with unique characters in a video game. After several sessions playing the game, the degree of increased activation within a speech-selective ROI in the left STS posterior to HG correlated with the degree of training success. Notably, this pattern did not emerge in a whole-brain analysis, and it may be the case that the creation of the speech-selective ROI may have eliminated the consideration of regions that would not respond to the speech vs. environmental sound contrast. Left posterior STS/STG activation has also been shown to correlate with training success in pitch pattern learning (Wong et al., 2007).

It is possible that the asymmetry between studies which have shown involvement of temporal regions in novel contrast sensitivity and those which have not may be attributed to the duration and/or intensity of training. Our study (Myers and Swan, 2012) employed only two 45-min sessions of training, whereas other studies have employed multiple intense training sessions. One proposal is that sensitivity to category-level information emerges early in the frontal lobe and only later is evident in temporal structures. This pattern would be consistent with a variety of proposals outside the language literature which suggest a shift from executive or category-level processing to sensory-based processing as expertise is gained (Ahissar and Hochstein, 2004; Nahum et al., 2008).

In order to address this question, we performed a replication of Myers and Swan (2012) in which we extended the training to ten 45-min sessions over 2 weeks (Myers et al., under review). Participants in this study were now trained to just distinguish dental and retroflex voiced stop consonants. Pre- and post-training scans were performed using the short-interval habituation design (Myers and Swan, 2012), and during scanning participants were asked to perform a pitch detection task in which they responded to high-pitched syllables on infrequent catch trials. Rather than search for areas which show global changes in activation as a function of training, we targeted regions which showed a differential sensitivity to between-category compared to within-category contrasts. Similar to other studies investigating categorical perception, the logic was that regions which showed sensitivity to the learned category structure following training could not be said to be influenced merely by changes in attention, motivation, or familiarity with the stimuli. At pre-test, only the left middle frontal gyrus showed differences in activation for between- compared to within-category stimuli. After training, activation differences were seen in a bilateral network including the left precentral gyrus, right and left STG, left IPL, and right insula. Importantly, both left and right posterior STG were shown to be correlated at post-test with participants' behavioral accuracy at post-test, suggesting that temporal activation resulting from 10 days of training was not only sensitive to the “categorical” nature of the stimuli (between vs. within) but also was predictive of learning.

### Individual variability in speech sound learning

Many of the above-mentioned studies have searched for the neural correlates of variability in the perception of non-native contrasts. Variability in non-native perception is evident not only in training studies, but also in the varying degrees of proficiency that second-language learners attain (e.g., Bradlow et al., 1997; Flege et al., 1999). Studies which have examined the neural correlates of these differences among learners have come to differing conclusions regarding the source of this variability. Diaz et al. (2008) report that poorer perceivers of non-native contrasts showed an attenuated MMN response compared to better perceivers. The source of the MMN was inferred from the latency and distribution across electrodes, and was hypothesized by the authors to be the frontal component. The authors interpreted this response as reflecting engagement of an attentional network in better perceivers, whereas the lack of difference in the temporal component reflected similar fidelity in acoustic-phonetic processing across better and poorer percievers. By contrast, a study by Raizada showed that the patterns of activation within the right Heschl's gyri of Japanese L2 learners were predictive of that population's ability to discriminate /l/ vs. /r/ contrasts (Raizada et al., 2010). In the end, it is likely that functional variation at multiple points in the phonetic processing stream contribute to differences in learning success, with some learners excelling because of superior acoustic processing, and others achieving success due to the appropriate deployment of auditory attention, for instance.

Individual differences in brain structure are also predictive of phonetic learning success. Work by Golestani et al. (2002) and Golestani and Pallier (2007) showed that better learners showed differences in brain morphology in the left HG and a greater leftwards asymmetry in parietal cortex which was evident in WM volume. This asymmetry may reflect more efficient or precise coding of acoustic information which is especially relevant in speech sound learning (although see Burgaleta et al., 2014 for a null finding relating brain morphology to speech perception abilities in a bilingual population). An advantage for processing the fine-grained aspects of sound might have surprising professional consequences as well. A unique study (Golestani et al., 2011) found that individuals who were employed as phoneticians showed differences in the morphology of left Heschl's gyrus compared to a control group. Of interest, there was also a correlation between the surface area and structure of the left pars opercularis and years of experience working as a phonetician, providing a hint that frontal differences in morphology may have arisen through experience-induced plasticity rather than from innate differences in brain structure.

This finding raises the question of whether experience learning a non-native phonetic contrast might actually induce structural changes in the brain. This type of plasticity is not unprecedented. Changes in brain morphology have been found following training on a variety of tasks (see Zatorre et al., 2012 for a review) and relevant for the current discussion, following a semester of intensive second-language learning (Stein et al., 2012). In our study of intensive non-native speech sound training (Myers et al., under review), changes in gray matter volume were seen in a region deep to the left supramarginal gyrus comparing pre-training scans to post-training scans. This same region is among the set of regions in which individual variation is associated with successful phonetic category learning (Golestani et al., 2007), and with individual differences in non-native sound production (Golestani and Pallier, 2007). Moreover, in our study, the coherence of white matter pathways (as measured by DTI) near the arcuate fasciculus in this same vicinity was seen to correlate with learning success, suggesting that the strength of frontal-to-posterior connections along the dorsal route contributes to non-native category learning. Taken together, these results suggest that even relatively short-term training can serve to strengthen connections that are necessary for non-native speech sound learning.

## A frontal to temporal route for phonetic category learning

The extant literature on non-native speech sound learning suggests that the long-term consequence of speech category training is the retuning of posterior temporal regions such that they show increased sensitivity to the dimensions of the learned speech sounds. Of note, this same region also shows sensitivity to phonetic category structure in native speech perception which is presumably acquired slowly over the course of development. Broadly speaking, this is consistent with most models of the neural bases of speech perception (Hickok and Poeppel, 2007; Rauschecker and Scott, 2009). However, data suggests that short-term adjustments to learned phonetic category structure may be seen first in the frontal lobe (Myers and Swan, 2012), and only after sustained or more intensive training do these same sensitivities appear in the posterior temporal lobe (e.g., Leech et al., 2009; Myers et al., under review). Moreover, individual training success correlates with the coherence of white matter pathways at pre-training (Myers et al., under review), in an area that is consistent with the dorsal stream route connecting posterior temporoparietal regions to frontal structures (Hickok and Poeppel, 2007). Of note, this frontal-to-temporoparietal route is not the only connection which has been shown to correlate with non-native training success. Resting-state functional connectivity before and after training reflects a decreased reliance on frontal-to-superior parietal connections after training (Ventura-Campos et al., 2013) which has been attributed to a decreased reliance on a “salience” network. Of note, Ventura-Campos and colleagues also show strong resting-state connectivity between the frontal operculum and the SMG, but this connectivity did not show any significant correlation with training success. The authors speculate that this lack of correlation may in part reflect the lower individual variability shown in the frontal-to-SMG connectivity findings.

This pattern of results leads us to propose that early learning of non-native speech categories in the context of explicit top-down information involves first feed-forward connections from posterior temporal cortex to ventrolateral prefrontal cortex (Garell et al., 2013), where acoustic representations access category-level (articulatory, phonological, or abstract) information (Figure 2). Categorical sensitivity to non-native speech sounds emerges first in the inferior frontal lobe as participants learn the boundaries through acoustic space which define functional categories. This allows for rapid learning of category boundaries without fundamentally reshaping neural sensitivity to low-level details of the signal. Over time, frontal-to-temporal feedback connections may serve as an error signal on auditory sensitivities to these speech sounds, reshaping the sensitivity of auditory association cortex. The view that frontal-to-temporal feedback signals may play a role in rapid auditory plasticity finds support from animal models (Winkowski et al., 2013), and human data suggests that stimulation of frontal sites may facilitate auditory perceptual learning (Sehm et al., 2013). We suggest that the process of retuning sensitivities in the temporal lobes unfolds more slowly, over the course of minimally several days of training or experience.

Notably, our findings suggest that learners can achieve at least moderate success in training without any detectable change in the responsiveness of the temporal lobes (Myers and Swan, 2012). One open question is whether training which only recruits frontal lobe is retained over time. It may be the case that temporal lobe encoding is actually necessary for long-term learning of the speech contrast (Myers et al., under review). It is also unknown whether short-term learning in the frontal lobes reflects a different perceptual status of the stimulus as compared to when this sensitivity emerges in the temporal lobes. For instance, it is possible that frontal encoding relies more heavily on domain-general systems for perceptual categorization whereas temporal encoding reflects a more genuine status of the stimuli as phonetic categories.

A system which allowed for rapid, on-the-fly adaptation to new phonetic category structure might present several advantages not only for learning new speech contrasts, but also for processing details of native language speech. As listeners, we are exposed to speech variants that differ significantly from our native language phonetic categories, for instance, in the case of foreign accents, yet we are also able to quickly adapt to non-standard speech sounds (Bradlow and Bent, 2008; Kraljic et al., 2008). A neural system which likewise showed rapid, contextually-sensitive flexibility to shift phonetic category boundaries would facilitate this kind of adaptation. At the same time, unconstrained flexibility in processing non-standard speech sounds could be disadvantageous—for instance, one's phonetic category boundaries should not be continuously perturbed by every exposure to a new talker or accent. As such, a separate neural system which shows more stable, slowly-adapting responses would be also advantageous.

Several testable predictions fall out of this type of model. First, if frontal-to-temporal feedback is necessary for non-native phonetic category learning, patients with frontal lobe pathology (e.g., individuals with Broca's aphasia) would have significant deficits in the acquisition and retention of new category information, while retaining sensitivities to native language phonetic category information learned pre-insult. Second, under the assumption that frontal systems are only engaged when category-level information is required for acquisition, it should be the case that incidental learning of phonetic categories, whether via sensitivity to statistical properties of the input (Hayes-Harb, 2007), or through other implicit methods (e.g., Lim and Holt, 2011; Vlahou et al., 2012) should be spared in this same population. Finally, if this frontal-to-temporal pathway is directed along the arcuate fasiculus, the coherence of this pathway should predict better speech sound learning at an individual level (see Myers et al., under review), and category training should be difficult for patients whose lesions implicate this pathway. Finally, as shown by Ventura-Campos et al. (2013), functional connectivity between frontal and posterior sites should inversely correlate with learning success as listeners transfer category-level learning to reshape perceptual sensitivities in the posterior temporal lobe.

## Conclusion

The model described here is motivated largely through training studies which have used explicit, metalinguistic tasks in order to induce phonetic category sensitivities. There is still much to learn regarding phonetic category acquisition. First, little is known regarding the mechanisms which support encoding of statistical/distributional information which may reshape sensitivities “for free” as listeners are passively exposed to a new language. In the visual and auditory (non-speech) modalities, evidence suggests that medial temporal lobe and subcortical structures, in particular the caudate, may play a crucial role in encoding statistical regularities in the input (e.g., Turk-Browne et al., 2009; Durrant et al., 2013). Yet it is unknown whether the same structures mediate statistical learning for non-native speech sounds. At least one study (Golestani and Zatorre, 2004) showed engagement of the caudate for non-native speech sounds after training, although this result was attributed by these authors to the role of the caudate in motor speech control rather than in statistical learning.

Relatedly, the process of learning a non-native contrast involves encoding speech sounds in memory, but also protecting these newly-learned sounds from interference from existing similar speech sounds in one's native language. Recent work from our lab (Earle and Myers, under review) suggests that consolidation during sleep plays a significant role in this process. Participants who learned a non-native speech contrast in the evening showed improvements in discrimination of this contrast after an overnight interval and 24 h after learning, whereas participants who learned the same contrast in the morning did not show retention of the contrast after sleep. A follow-up suggested that the morning group's failure to retain the contrast was due to interference from exposure to similar native-language speech sounds over the course of the day. Taken together, this evidence suggests that (a) sleep plays a stabilizing role in the perceptual learning of speech sounds and (b) interference before sleep can serve to disrupt perceptual learning. This finding joins a literature on perceptual learning of synthetic speech sounds (Fenn et al., 2003, 2013) and on lexical learning which point to a crucial role for sleep in either abstracting away from the episodic details of the input, or to protection of learning from decay. While the neural bases of sleep-related consolidation for speech sounds have yet to be investigated, following a complementary systems memory framework (McClelland et al., 1995; O'Reilly and Rudy, 2001), one might predict that immediate encoding of novel speech sounds would implicate the hippocampus, while the overnight interval would serve to transfer this learning to cortical systems (e.g., Davis et al., 2009). This hippocampal-to-cortical transfer is thought to support abstraction from the episodic details of the signal to a more abstract representation of the input.

Perhaps most importantly it has yet to be determined whether second-language learning in immersion or in the classroom induces the same types of neural responses observed here. To fully understand the boundaries of plasticity in adult phonetic category learning, future research will need to be directed at these topics.

### Conflict of interest statement

The author declares that the research was conducted in the absence of any commercial or financial relationships that could be construed as a potential conflict of interest.
